# Metabolic Liver Disease and Cancer Transition: Central Role of the FBP1 Regulatory Axis

**DOI:** 10.1002/mco2.70347

**Published:** 2025-08-15

**Authors:** Zhengtao Liu, Jun Xu, Shusen Zheng

**Affiliations:** ^1^ Key Laboratory of Artificial Organs and Computational Medicine in Zhejiang Province Shulan International Medical College Zhejiang Shuren University Hangzhou China; ^2^ NHC Key Laboratory of Combined Multi‐Organ Transplantation Key Laboratory of the Diagnosis and Treatment of Organ Transplantation First Affiliated Hospital School of Medicine Zhejiang University Hangzhou China; ^3^ Department of Surgery Division of Hepatobiliary and Pancreatic Surgery First Affiliated Hospital School of Medicine Zhejiang University Hangzhou China

1

In the recent article published in Nature [1], Gu and colleagues investigated the role of fructose‐1,6‐bisphosphatase 1 (FBP1) in hepatocellular carcinoma (HCC) originating from senescent hepatocytes in metabolic dysfunction‐associated steatohepatitis (MASH). They found FBP1, a p53 target, elevated in senescent‐like MASH hepatocytes but suppressed in most HCCs via promoter hypermethylation and proteasomal degradation, identifying an FBP1‐p53‐AKT‐NRF2 metabolic switch that reverses senescence and enables DNA damage‐induced mutations driving transformation from MASH to liver cancer.

MASH is a progressive liver disease characterised by steatosis, inflammation, and fibrosis, which can eventually lead to cirrhosis and liver failure. Accordingly, HCC is increasingly recognised as a major complication of MASH even in the absence of cirrhosis. Followed by global increments in obesity and metabolic syndrome, the mechanisms underlying the MASH‐driven HCC have recently become a key research focus for further effective prevention and targeted therapies [2, 3]. Despite progress achieved, the MASH‐HCC transformation is still a conundrum with varied candidate pathways across two different disease statuses [2].

Liver aging refers to the progressive decline in liver function and regenerative capacity due to cellular senescence, driven by oxidative stress, mitochondrial dysfunction, and chronic inflammation [4]. The authors revealed MASH induced p53‐dependent hepatocyte senescence in alliance with hypernutrition‐induced DNA breaks. However, cellular senescence had protective effects on tumorigenesis by reducing cellular proliferation and DNA damage mediated by cell cycle inhibitors like p21^Cip1^ and p16^Ink4a^. MASH cannot directly increase the HCC susceptibility by inducing hepatocyte senescence. MASH is dynamic, where key genetic regulation is not static throughout the whole process. The authors hypothesized that certain specific switches might promote malignant transformation from inflammatory hepatocytes to tumor cells.

The FBP1 gene is a target of p53, which encodes fructose‐1,6‐bisphosphatase 1, a key enzyme in gluconeogenesis. In the MASH context, FBP1 expression was elevated in response to metabolic stress and DNA damage, contributing to hepatocyte senescence.

Based on human samples and the mouse MASH model, the authors confirmed: 1. FBP1 functioned as a tumor suppressor by inducing cellular senescence and inhibiting cell proliferation. It was upregulated in senescent MASH hepatocytes, preventing cancer by maintaining a senescent state. 2. FBP1 was downregulated in progenitor HCC cells via promoter hypermethylation and proteasomal degradation, correlated with activation of oncogenic NRF2 and AKT pathways. 3. FBP1 regulated glucose metabolism by stimulating gluconeogenesis and suppressing glycolysis. FBP1 knockout mice exhibit accelerated HCC progression with increased tumor burden and markers. This is characterized by enhanced hepatocyte proliferation and fibrosis, likely due to the metabolic stress and activation of oncogenic pathways in the absence of FBP1.

Meanwhile, NRF2 encodes a transcription factor that regulates the antioxidants and metabolic genes. In HCC progenitor cells, NRF2 was up‐regulated due to metabolic stress and DNA damage. DNA damage‐activated ATM/ATR signaling indirectly affected NRF2 activity in HCC, likely via phosphorylating the NRF2 upstream regulators. NRF2 activation promoted ERK1/2 phosphorylation by inducing the production of growth factors to trigger the activating signaling cascade. Otherwise, NRF2‐induced FBP1 degradation relieved AKT inhibition and enhanced the activation of upstream AKT‐signaling kinases to promote its phosphorylation to enhance cell proliferation and subsequent HCC progression. FBP1 suppressed the NRF2 activity by inhibiting the Ser9 phosphorylation of GSK3β, which led to increased NRF2 degradation and maintained its lower activity. Meanwhile, NRF2 led to FBP1 degradation. And activated NRF2 promoted the FBP1 degradation through mechanisms involving ERK signaling, which in turn removed the inhibitory effect of FBP1 on NRF2, creating a positive feedback loop to enhance the NRF2 activity. This interplay between FBP1 and NRF2 is critical in the context of metabolic stress and DNA damage, which determines the fate of hepatocytes to a state of senescence or proliferation, ultimately influencing the progression from MASH to HCC. Hence, the authors proposed the “FBP1–NRF2–AKT–p53” regulatory axis, which is instrumental in disease progression. FBP1 was down‐regulated in precancerous tissues, thereby triggering NRF2 activation. Subsequently, NRF2 not only promoted the FBP1 degradation but also accelerated the p53 degradation by enhancing AKT activation. This coordinated event cascade ultimately supported the reversal from senescence to proliferation in progenitor cells, facilitating the “MASH‐HCC” transition (Figure 1).

**FIGURE 1:**
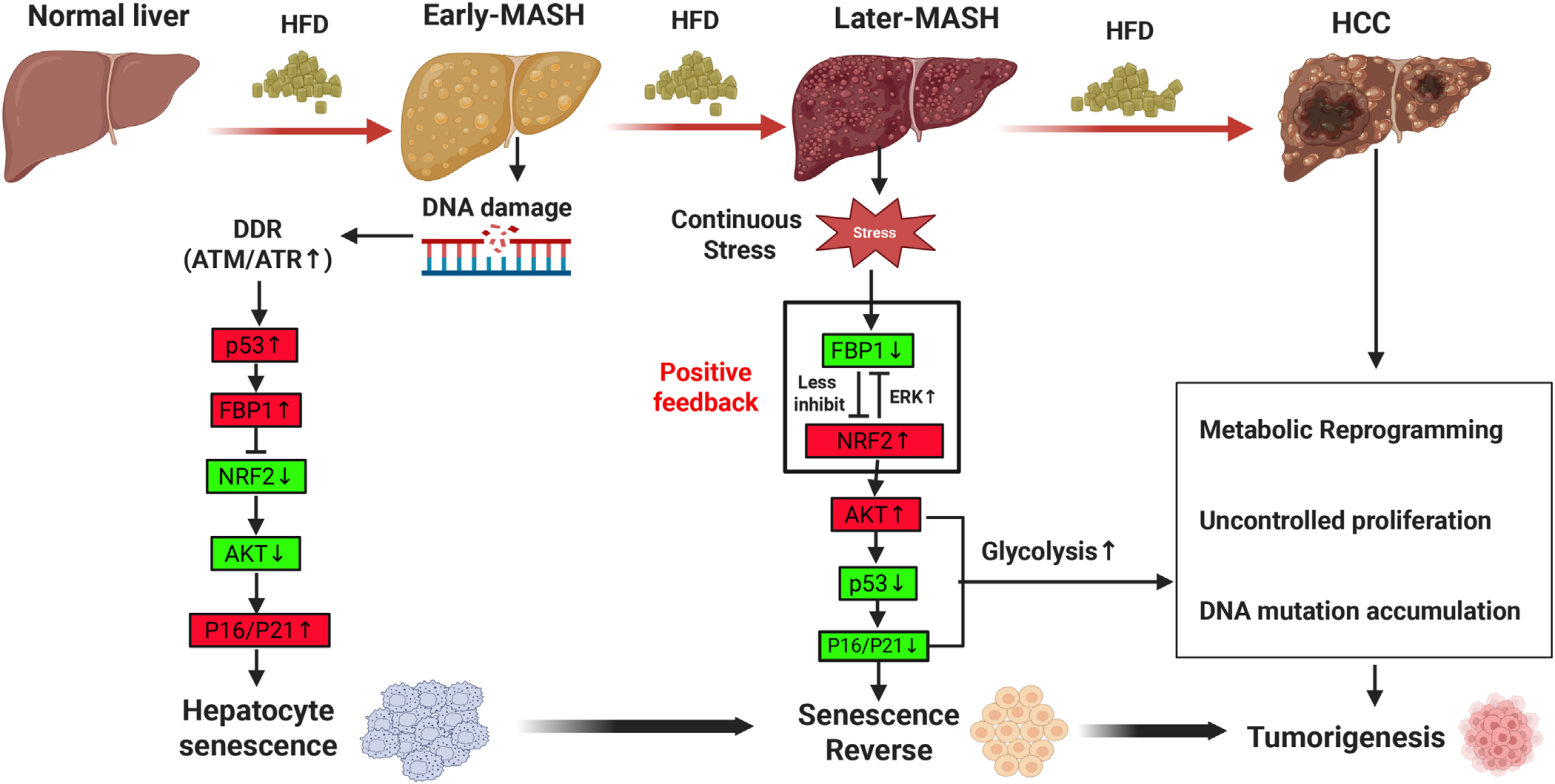
HFD‐driven progression from MASH to HCC: mechanisms involving FBP1‐NRF2‐AKT‐p53 regulatory axis. This figure illustrates the sequential pathological progression from normal liver to hepatocellular carcinoma (HCC) under continuous high‐fructose diet (HFD) stimulation, highlighting key molecular events and phenotypic transitions. Normal liver to Early‐Metabolic dysfunction‐associated steatohepatitis (MASH): Persistent HFD exposure induces DNA damage and metabolic stress, activating the DNA damage response (DDR). This triggers p53 upregulation, which promotes FBP1 expression. FBP1 inhibits AKT activation and maintains low NRF2 activity, thereby inducing hepatocyte senescence via upregulated P16/P21 and suppressing malignant transformation. Early‐MASH to Later‐MASH: With prolonged HFD stress, FBP1 expression is downregulated, relieving inhibition on NRF2 and AKT. A positive feedback loop is initiated: activated NRF2 further reduces FBP1 via ERK‐mediated degradation, while AKT hyperactivation promotes p53 degradation. This leads to senescence reversal as P16/P21 decreases, allowing hepatocytes to escape growth arrest. Later‐MASH to HCC: Dysfunction of the FBP1‐NRF2‐AKT‐p53 axis drives metabolic reprogramming by enhanced glycolysis, DNA mutation accumulation, and uncontrolled hepatocyte proliferation, which ultimately results in final tumorigenesis. The graphical model was generated by applying the Biorender website (https://biorender.com/). Green boxes indicate downregulation; red boxes indicate upregulation.

Translational potentials and implications for other research are mainly reflected as follows: First, this study has delineated the MASH‐HCC progression by distinct phases. The accumulation of inflammation led to perturbations in the regulatory axis for liver senescence, which can cause irreversible HCC development. Further investigation on factors influencing this regulatory switch and the development of targeted interventions could help suppress the cancer progression from MASH. Second, this article primarily focused on the somatic regulation axis, but with implications for other cells, both in MASH and HCC, like macrophages, T‐cells, and dendritic cells. Considering the carcinogenesis of MASH is not an immediate process, single‐cell RNA sequencing and in‐depth mechanistic analysis on samples at different stages are of great significance in understanding the specific cells in the “MASH‐HCC” transition. Third, tumor characteristics encompass aspects not only including cell proliferation but also apoptosis, angiogenesis, metastasis, immune evasion, metabolic reprogramming, genomic instability, inflammation, evasion of growth suppression, resistance to cell death, tumor microenvironment remodeling, and epigenetic alterations [5]. Exploration of this regulatory axis on tumor malignancy will enhance our understanding of the MASH‐related oncogenic regulatory axis. Fourth, future work should investigate whether the FBP1‐centered metabolic axis operates in other disorders like diabetes, and systematically map FBP1 expression heterogeneity across diverse tissues to fully assess its systemic metabolic consequences. Fifth, given NRF2's dual role in cytoprotection and oncogenesis, efforts should prioritize the selective NRF2 inhibitors that can block its tumor‐promoting hyperactivation while preserving antioxidant function.

We emphasize the immediate clinical value of translating the FBP1‐NRF2‐AKT‐p53 axis into actionable therapeutics: low‐serum‐FBP1/high‐NRF2/low‐p53 signatures could guide early intervention, while parallel preclinical pipelines for FBP1 agonists, selective NRF2 inhibitors, and DNA‐damage‐response agents should be prioritized to convert this foundational discovery into therapies against MASH‐driven HCC.

In conclusion, this study provided critical insights into the mechanisms underlying the transition from MASH to HCC, emphasizing the role of FBP1 and its interplay with NRF2, AKT, and p53 in hepatocyte senescence and tumorigenesis. The identification of the “FBP1‐NRF2‐AKT‐p53” axis highlighted a key metabolic and signaling switch governing the hepatocyte fate with potential benefits for therapeutic intervention. By elucidating the dynamic regulatory mechanisms, this article not only advanced understanding of MASH‐driven HCC progression but also laid the groundwork for future investigations into targeted strategies to prevent or mitigate this malignant transformation.

## Author Contributions

Z.T.L. conceived and designed the research; J.X. interpreted the data; Z.T.L. wrote the manuscript; S.S.Z. reviewed the manuscript. All authors have read and approved the final version of the manuscript.

## Conflicts of Interest

The author declares no conflicts of interest.

## Ethics Statement

The author has nothing to report.

## Data Availability

The authors have nothing to report.
